# Urinary exosomal microRNAs as predictive biomarkers for persistent psychotic-like experiences

**DOI:** 10.1038/s41537-023-00340-5

**Published:** 2023-03-11

**Authors:** Yasufumi Tomita, Kazuhiro Suzuki, Syudo Yamasaki, Kazuya Toriumi, Mitsuhiro Miyashita, Shuntaro Ando, Kaori Endo, Akane Yoshikawa, Koichi Tabata, Satoshi Usami, Mariko Hiraiwa-Hasegawa, Masanari Itokawa, Hideya Kawaji, Kiyoto Kasai, Atsushi Nishida, Makoto Arai

**Affiliations:** 1 Schizophrenia Research Project, Department of Psychiatry and Behavioral Sciences, Tokyo Metropolitan Institute of Medical Science, Tokyo, Japan; 2 Department of Computational Biology and Medical Sciences, Graduate School of Frontier Sciences, The University of Tokyo, Tokyo, Japan; 3 Department of Psychiatry, Shinshu University School of Medicine, Matsumoto, Japan; 4 Department of Community Mental Health, Shinshu University School of Medicine, Matsumoto, Japan; 5 Unit for Mental Health Promotion, Research Center for Social Science and Medicine, Tokyo Metropolitan Institute of Medical Science, Tokyo, Japan; 6 Department of Neuropsychiatry, Graduate School of Medicine, The University of Tokyo, Tokyo, Japan; 7 Department of Psychiatry and Behavioral Science, Juntendo University Graduate School of Medicine, Tokyo, Japan; 8 Department of Psychiatry and Behavioral Sciences, Graduate School of Medical and Dental Sciences, Tokyo Medical and Dental University, Tokyo, Japan; 9 Center for Research and Development on Transition from Secondary to Higher Education, The University of Tokyo, Tokyo, Japan; 10 Department of Evolutionary Studies of Biosystems, The Graduate University for the Advanced Studies, SOKENDAI, Hayama, Japan; 11 Research Center for Genome & Medical Sciences, Tokyo Metropolitan Institute of Medical Science, Tokyo, Japan; 12 International Research Center for Neurointelligence, University of Tokyo Institutes for Advanced Study, University of Tokyo, Tokyo, Japan

**Keywords:** Biomarkers, Psychosis

## Abstract

Psychotic-like experiences (PLEs) occur occasionally in adolescence and mostly disappear with increasing age. Their presence, if persistent, is considered a robust risk factor for subsequent psychiatric disorders. To date, only a few biological markers have been investigated for persistent PLE prediction. This study identified urinary exosomal microRNAs that can serve as predictive biomarkers for persistent PLEs. This study was part of a population-based biomarker subsample study of the Tokyo Teen Cohort Study. A total of 345 participants aged 13 (baseline) and 14 (follow-up) years underwent PLE assessments by experienced psychiatrists using semi-structured interviews. We defined remitted and persistent PLEs based on longitudinal profiles. We obtained urine at baseline and the expression levels of urinary exosomal miRNAs were compared between 15 individuals with persistent PLEs and 15 age- and sex-matched individuals with remitted PLEs. We constructed a logistic regression model to examine whether miRNA expression levels could predict persistent PLEs. We identified six significant differentially expressed microRNAs, namely hsa-miR-486-5p, hsa-miR-199a-3p, hsa-miR-144-5p, hsa-miR-451a, hsa-miR-143-3p, and hsa-miR-142-3p. The predictive model showed an area under the curve of 0.860 (95% confidence interval: 0.713–0.993) for five-fold cross-validation. We found a subset of urinary exosomal microRNAs that were differentially expressed in persistent PLEs and presented the likelihood that a microRNA-based statistical model could predict them with high accuracy. Therefore, urine exosomal miRNAs may serve as novel biomarkers for the risk of psychiatric disorders.

## Introduction

Psychotic-like experiences (PLEs) are psychotic symptoms that may occasionally occur during adolescence in the absence of illnesses^1^. According to some meta-analyses, the prevalence of PLEs is estimated at 5–17% in the general population^1–4^. The prevalence varies by age but it also depends on the method used to assess PLE^5–9^. Most PLEs are transient and disappear with increasing age; however, some PLEs persist till late adolescence^3,10,11^. PLE symptoms are not always accompanied by illnesses or distress, and the same is true for persistent PLE^4^. However, persistence of PLEs is known to increase susceptibility to psychotic disorders and elevate the risk of schizophrenia, depression, and suicidal tendencies^4,12–15^. In particular, persistent and distressing PLEs show poor psychopathological prognoses later in life^16^. Thus, PLEs are regarded as early intervention targets for psychiatric disorders ^12,17^. For a more selective intervention, it is necessary to predict whether PLEs will persist^11,15^. However, to the best of our knowledge, only a few biological markers have been investigated for such a prediction^18^. Therefore, the development of biomarkers for persistent PLEs could contribute to the early detection and intervention of psychiatric disorders.

Recently, exosomal miRNAs have received a lot of attention as biomarkers for psychiatric disorders^19–21^. miRNAs are short non-coding RNAs that function as post-transcriptional regulators of gene expression^22^. A single miRNA can regulate expressions of multiple genes by binding complementarily to the 3′ untranslated region of mRNAs, inhibiting mRNA translation, promoting degradation of the corresponding mRNA, and ultimately reducing its protein expression^23^. No studies on the relevance of miRNAs and PLE have been published. However, miRNA expression was reported to change in the first episode psychosis, suggesting that miRNA involved in the onset of psychiatric disorders^24,25^. Considering persistent PLE as a predictor of psychiatric disorders, miRNA expression in persistent PLE should be investigated.

Exosomal miRNAs are contained in nanosized extracellular vesicles called “exosomes” and are stably detected in plasma, serum, cerebrospinal fluid, urine, and other body fluids^26^. Therefore, exosomal miRNAs in liquid biopsies are easily accessible in living patients and recognized as promising biomarkers for psychiatric disorders^27,28^. In liquid biopsy, urine has some advantages compared to blood. Owing to its non-invasive sampling method, urine is ideal for liquid biopsy of adolescents as compared to blood. This sampling is more acceptable by adolescents and can be repeated regularly, as often as required. Moreover, a previous study suggests that urine exosomes are in part, derived from brain tissue^29^. Urinary miRNAs and exosomes are available as biomarkers for diagnosing tumors of the central nervous system and Alzheimer’s disease^30,31^. Considering these observations, some miRNAs in urinary exosomes may be derived from neural cells and can reflect the pathophysiology of the brain.

Here, we examined whether urinary exosomal miRNA expression profiles in adolescents can be used as biomarkers for predicting the persistence of PLEs.

## Patients and methods

### Study design and participants

The study examined biological markers, including urinary exosomal miRNAs, and assessed PLEs using semi-structured interviews by expert psychiatrists.

**Fig. 1:**
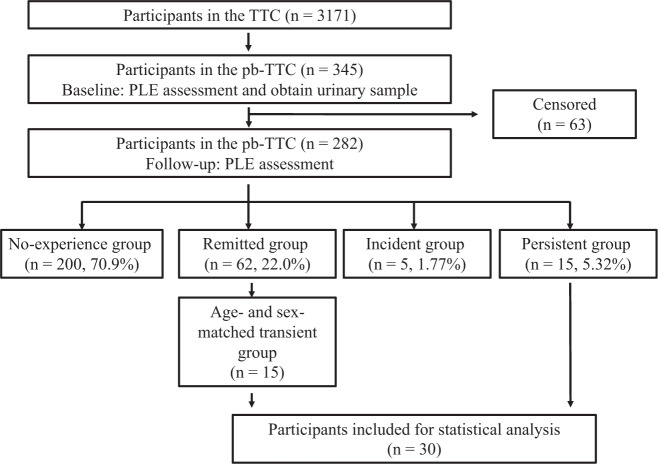
Flowchart of participant recruitment in this study. Flowchart for participant classification is shown. PLE psychotic-like experience, pb-TTC population-based Tokyo Teen Cohort, TTC Tokyo Teen Cohort.

The current study was part of a population-based biomarker subsample study of the Tokyo Teen Cohort Study^32^ (TTC; http://ttcp.umin.jp/) (pb-TTC), in which 345 adolescents aged (mean [standard deviation, SD] 13.5 [0.6] years and their caregivers (mainly mothers) were included. Participants in the pb-TTC were recruited from a larger sample of the TTC project, a large-scale population-based birth cohort study conducted in the Tokyo metropolitan area, wherein 3171 adolescent–caregiver dyads were present. No significant differences in age, sex, or socioeconomic status (*p* > 0.05) were found between the pb-TTC subsample and the TTC population. We longitudinally followed up with the pb-TTC subsample after a year. Of the 345 participants in the baseline survey, 282 participants (82%) completed the follow-up survey. We compared the 15 cases in the persistent group with 15 age- and sex-matched participants with remitted PLE (Fig. 1). The PLE groups are defined in the next section.

Written informed consent was obtained from each participant and their primary caregiver before participation in the study. This study was approved by the Research Ethics Committee of the Tokyo Metropolitan Institute of Medical Sciences.

### Data collection

Briefly, participants were assessed for PLE at age 13 and exactly 1 year later using semi-structured interviews based on self-report questionnaires. In this study, only children with PLE within the past 6 months from the time of assessment were identified as PLE-positive. First, they were asked to respond to a brief self-report questionnaire. This questionnaire included the Adolescent Psychotic-Like Symptom Screener (APSS), which can screen the general adolescent population for PLEs^33^. The APSS comprises seven items concerning PLEs as follows: hallucinations (visual and auditory) and delusions (mind reading, reference, being spied on, being in control, and grandiose ability). For each question, there were three possible answers: “No, never,” “Maybe,” and “Yes, definitely.” Second, to assess PLE, experienced psychiatrists (S.A., M.M., and K.S.) interviewed all patients by looking at their APSS responses about the details of their experiences and the timing of the experience. If the child answered “Maybe” or “Yes, definitely” to any of the seven items of the APSS, the responses were cross-examined in a semi-structured interview using each of the seven related symptoms from the Scales for the Assessment of Positive Symptoms to obtain an observer’s assessment of the symptoms. In particular, the psychiatrists asked for details to determine if they were PLEs for those with the “Maybe” response. We recorded each positive symptom episode and its frequency in the previous 6 months. Interviewers also checked whether positive symptoms were experienced when the adolescent was in a hypnagogic and hypnopompic state, had a fever, or had been drinking alcohol or using drugs. Third, after each interview, three psychiatrists held consensus meetings (S.A., M.M., and K.S.) to review each interview record. Participants were rated as having “definite” PLEs without attributions if there was consensus among all three psychiatrists. The baseline PLEs were assessed when patients were 13 years old. All patients underwent follow-up PLE assessment just after a year.

We defined the following four groups based on the longitudinal profiles of PLEs across the two-time points (baseline and follow-up): the no-experience group (i.e., individuals without PLEs at both time points), the remitted group (i.e., individuals with PLEs at baseline and without PLEs at follow-up), the incident group (i.e., individuals without PLEs at baseline and with PLEs at follow-up) and the persistent group (i.e., individuals with PLEs at both time points). The participants for miRNA-seq analysis showed concordant results between seif-report questionnaires and interviews.

### Sample collection, exosome isolation, miRNA extraction, and miRNA sequencing

We obtained exosomal miRNAs from the urine samples of participants. Urinary exosomal miRNAs are useful tools for easy, non-invasive sampling, especially in adolescents. Urine samples were collected from all participants in the morning on the day of the interview at 13 years of age and stored at 4 °C until further processing, which comprised centrifugation at 3000 × *g* for 10 min. The urine supernatant was transferred into a new tube and stored at −80 °C for analysis at a later stage.

miRNA expression data analysis encompassing all steps of high-throughput next-generation sequencing with the NovaSeq 6000 platform (Illumina, San Diego, CA, United States) was conducted by Takara Bio Inc. (Kusatsu, Japan). Briefly, urinary exosomes were obtained from ~5–10 ml of urine using a miRCURY Exosome Cell/Urine/CSF Kit (QIAGEN, Hilden, Germany), and RNA was obtained from the extracted exosome sample using a miRNeasy Micro kit (QIAGEN). The quality of the extracted RNA was evaluated using a Bioanalyzer 2100 (Agilent, Santa Clara, CA, United States), and a clear peak was observed in the size of the small RNA (<200 nt). A miRNA-Seq library was prepared using high-quality RNA with a yield of 2 ng (400 pg/μl) using a QIA-seq miRNA Library Kit (QIAGEN).

Sequencing of the miRNA library resulted in 1,409,409,273 sequences (29,362,693 sequences per donor on average). With over ten thousand reads on average, the quantification ability is likely to be saturated. The sequences were aligned with miRbase release 22.1, using the CLC Genomics Workbench (v12.0.3) Biomedical Genomics Analysis Plugin (v1.2.1), after trimming the adaptor sequences. Counts of unique molecular identifiers (UMIs) included in the reads that matched individual miRNAs were subjected to subsequent miRNA expression analysis.

### Analysis of miRNA profiles

Counts of UMIs were normalized as transcripts per million using the run-length encoder method^34^ in the bioconductor/edgeR package (version 3.30.3). Differential analysis was performed with the likelihood ratio test on edgeR by comparing the group of adolescents with persistent PLEs and those with remitted PLEs. The full model had group and sex as variables, while the reduced model only had sex as a variable. *p* values were adjusted using the Benjamini–Hochberg false discovery rate procedure.

### Statistical analysis

Statistical analysis was performed using R 4.0.4 (released on 02-15-2021). Logistic regression models were constructed to estimate the probability of persistent PLEs using the glm function in R based on 6 miRNAs expressions with significant differences, where their normalized expression levels were used as explanatory variables. We evaluated the predictive performance of the models using the area under the receiver operating characteristic (ROC) curve. All areas under the curve (AUCs) reported by the ROC function in the “pROC” R package and 95% confidence interval (CI) of AUC were presumed in the DeLong method.

### Cross-validation

To examine the degree of overfitting of the prediction model to our dataset, we validated the predictive model using five-fold cross-validation instead of using an independent dataset. This cross-validation procedure was performed for the six miRNAs panel multivariate prediction models, in which each covariate was assigned a separate regression coefficient. All AUCs reported by the ROC function in the pROC R package and 95% CI of AUC were presumed using the DeLong method. The average AUCs of covariates were calculated as the arithmetic mean.

### Pathway enrichment analysis

The DIANA-mirPath v.3 tool was used for pathway analyses. We used microT-CDSv5.0 to identify miRNA-mRNA interactions and probe the Kyoto Encyclopedia of Genes and Genomes (KEGG) for pathway analysis.

## Results

### Demographic data and clinical characteristics of participants

We assessed PLEs in 345 adolescents at two-time points (details are provided in the “Patients and methods” section). As a result of PLE assessment, there were 200 in the no-experience groups (70.9%), 62 in the remitted groups (22.0%), 5 in the incident groups (1.77%), and 15 in the persistent groups (5.32%) in this population-based subsamples (Fig. 1). In the baseline assessment, 345 individuals participated and 92 individuals had PLEs (26.7%). In the follow-up assessment, 282 individuals participated and 20 individuals had PLEs (7.09%). In total, 15 adolescents with persistent PLEs who were assessed as experiencing PLEs at both baseline and follow-up, and 15 adolescents with remitted PLEs who were assessed as experiencing PLEs at baseline participated in the downstream analysis (Fig. 1) were included. The demographic characteristics of the participants are presented in Table 1. No significant differences were observed in terms of age or sex between the two groups.

**Table 1:** Demographic characteristics of the study participants (*n* = 30).

	Persistent	Remitted	*p*
Participants, *n*	15	15	
Male/Female, *n*	9/6	9/6	
Race (Asian), *n*	15	15	
Age at baseline, month, mean ± SD	167 ± 5.81	166 ± 5.27	0.729

Demographic data were compared between the persistent and remitted groups. Statistical comparisons were performed using Welch’s *t*-test.

*SD* standard deviation.

### Quantification of miRNA expressions

**Fig. 2:**
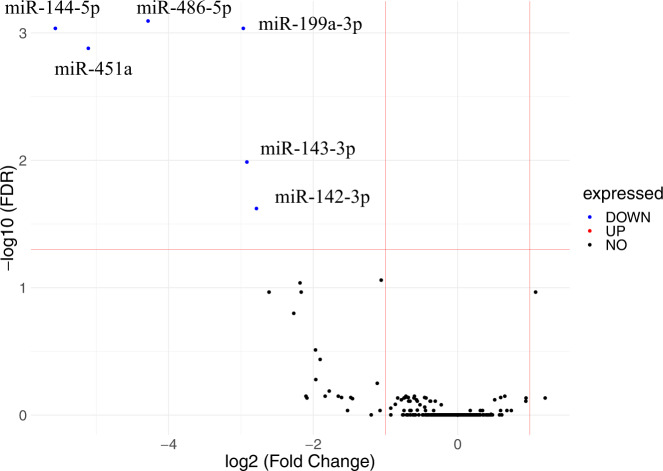
miRNA expression changes in the urinary exosome of adolescents with persistent PLEs. The expression of six miRNAs changes significantly in persistent PLEs. The fold change (persistent vs. remitted) of each miRNA is plotted against its corrected *p* value; blue circles represent miRNAs with FDR < 0.05, and log2 (Fold Change) < −1.0. Red lines represent the threshold of significant difference with FDR < 0.05, and log2 (Fold Change) > 1.0. FDR false discovery rate, PLE psychotic-like experience, miRNA microRNA.

Among the 2631 miRNAs found in all participants, 427 miRNAs were expressed over 10 transcripts per million (TPM) in at least three out of all subjects and were chosen for downstream analysis. Among them, six miRNAs (hsa-miR-486-5p, hsa-miR-199a-3p, hsa-miR-144-5p, hsa-miR-451a, hsa-miR-143-3p, and hsa-miR-142-3p) were differentially expressed between the persistent and remitted groups with a false discovery rate (FDR) < 5% and changes larger than two-fold, which was statistically significant (Fig. 2 and Table S1). All six differentially expressed miRNAs were downregulated in subjects with persistent PLEs, and none of them were upregulated.

### Prediction model

**Fig. 3:**
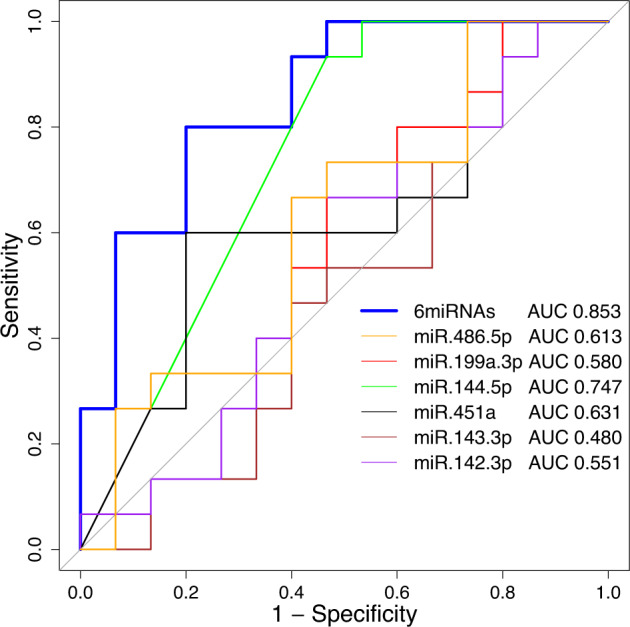
Receiver operating characteristic (ROC) curve of the six differentially expressed miRNAs and six miRNAs combined. Different colors denote the AUC for each miRNA. AUC area under the curve, ROC receiver operating characteristic, miRNA microRNA.

We built logistic regression models to predict PLEs at 14 years old using miRNA expression data obtained at 13 years old. In a logistic regression model of the combined six miRNAs with significant differences as explanatory variables, the model showed an AUC of 0.853, whereas univariate logistic models using a single miRNA as an explanatory variable showed an AUC ranging from 0.48 to 0.747 (Fig. 3).

### Validation of the performance of the six miRNAs

We examined variations in the performance of the six miRNAs and performed five-fold cross-validation, in which we iterated the following process five times: a logistic model was built using 80% of the participants, and its performance was assessed using the remaining 20%. The average AUC was 0.847 (95% CI 0.690–0.994). The sensitivity, specificity, and accuracy were 89.5 ± 10.7% (mean ± SD), 74.1 ± 13.3%, and 82.5 ± 3.12%, respectively, and the Youden index was 0.451 ± 0.0974.

### Pathway enrichment analysis

The Diana mirPath v.3 web-based computational tool was used for the KEGG pathway enrichment analysis. KEGG pathway enrichment analysis (*p* < 0.05) showed that the six differentially expressed miRNAs were associated with 21 different pathways in dopaminergic synapse and ECM-receptor interaction, as shown in Table 2.

**Table 2:** Pathway enrichment analysis of target genes.

KEGG pathway	Adjusted *p* value	Number of genes	Number of miRNAs
Thyroid hormone signaling pathway (hsa04919)	1.38E−05	26	5
ECM-receptor interaction (hsa04512)	0.000361	13	4
Mucin type O-Glycan biosynthesis (hsa00512)	0.0165	4	3
Sphingolipid signaling pathway (hsa04071)	0.0165	22	4
PI3K-Akt signaling pathway (hsa04151)	0.0165	51	6
Dorso-ventral axis formation (hsa04320)	0.0264	9	5
Pancreatic cancer (hsa05212)	0.0267	12	4
Cholinergic synapse (hsa04725)	0.0323	20	4
Glioma (hsa05214)	0.0323	12	4
Melanoma (hsa05218)	0.0323	15	4
cGMP-PKG signaling pathway (hsa04022)	0.0323	28	5
Phosphatidylinositol signaling system (hsa04070)	0.0323	13	5
Adrenergic signaling in cardiomyocytes (hsa04261)	0.0323	22	5
Estrogen signaling pathway (hsa04915)	0.0323	16	5
Proteoglycans in cancer (hsa05205)	0.0323	24	5
ErbB signaling pathway (hsa04012)	0.0323	18	6
mTOR signaling pathway (hsa04150)	0.0323	13	6
Dopaminergic synapse (hsa04728)	0.0323	24	6
Aldosterone-regulated sodium reabsorption (hsa04960)	0.0399	10	4
Ras signaling pathway (hsa04014)	0.0442	31	5
TGF-beta signaling pathway (hsa04350)	0.0471	12	5

Significant biological pathways were based on the target genes of the six miRNAs. The number of genes/miRNAs represents the number of target genes or miRNAs relative to the pathway. Target gene prediction was based on the microT-CDS database. The micro-T threshold was set to 0.8.

*KEGG* Kyoto Encyclopedia of Genes and Genomes, *miRNA* microRNA.

## Discussion

In this study, we quantified the expression levels of urinary exosomal miRNAs using deep sequencing and compared the expression profiles of adolescents with persistent and remitted PLEs albeit in a small sample size. We found that six miRNAs (hsa-miR-486-5p, hsa-miR-199a-3p, hsa-miR-144-5p, hsa-miR-451a, hsa-miR-143-3p, and hsa-miR-142-3p) were significantly decreased in adolescents with persistent PLEs compared with those with remitted PLEs.

Furthermore, we found that a subset of six miRNAs predicted whether PLEs would persist. Collectively, the subset had a significantly higher predictive ability than the individual miRNAs, showing sufficient performance as a novel biomarker to predict if PLEs persist. In addition, five-fold cross-validation suggested that our model did not overfit. Notably, however, a small sample size was used in the training and test sets.

To the best of our knowledge, this is the first study to suggest that miRNAs in urinary exosomes are useful for predicting persistent PLEs. Recent studies have reported that persistent PLEs are associated with the risk of psychiatric disorders; therefore, persistent PLEs are important targets for early intervention to prevent subsequent development^35,36^. Except for fingertip advanced glycation end products^18^, only a few biological risk factors for persistent PLEs have been found. Thus, our findings provide new insights into the early detection of PLEs with a high risk of transitioning to psychiatric disorders in clinical settings.

Three of the six miRNAs have been reported in several case-control studies of psychiatric disorders in plasma or serum samples. Hsa-miR-144-5p was upregulated in patients with schizophrenia^37^ and hsa-miR-144-5p, hsa-miR-451a, and hsa-miR-143-3p were downregulated in patients with major depressive disorder (MDD)^21,38–40^. Hsa-miR-199a was related to cognitive impairment^41^ and neuritin expression^42^. Neuritin has also been implicated in schizophrenia and MDD^43^. Thus, the functions of these miRNAs may be related to persistent PLEs and the subsequent development of psychiatric disorders. Additionally, we compared miRNA expressions between individuals without and with PLEs at baseline. We found 5 miRNAs (hsa-miR-888-5p, hsa-miR-199a-3p, hsa-miR-143-3p, hsa-miR-378d, and hsa-miR-891a-5p) with significantly altered expression (Fig. S1). Among them, hsa-miR-143-3p and hsa-miR-199a-3p had significantly altered expression in persistent PLE, suggesting that these are associated not only with the persistence of PLE but also with its presence.

In the enrichment pathway analysis of the target genes of the six miRNAs, several notable pathways were identified. Eleven pathways were associated with schizophrenia, including extracellular matrix (ECM) receptor interaction, sphingolipid signaling, PI3K-Akt signaling, cholinergic synapse, phosphatidylinositol signaling system, estrogen signaling pathway, ErbB signaling pathway, mTOR signaling pathway, dopaminergic synapse, ras signaling pathway, and TGF-beta signaling pathway^44–54^, whereas seven pathways were associated with MDD (Table S2).

hsa-miR-142-3p and hsa-miR-199a-3p identified in the present study have been experimentally shown to regulate KIF5B^55,56^. KIF5B is a motor protein in the dopaminergic synapse pathway and a member of the kinesin superfamily (KIF)^57,58^. KIF5B working with DISC1 is associated with the axonal transport of synaptic cargoes^59^ and is involved in neuron dopamine metabolism^58^. Some studies suggest that dopamine dysregulation is a mechanism underlying PLE^60,61^. Moreover, abnormalities in KIF proteins are reportedly associated with schizophrenia^62^. Although the association between KIF proteins and PLE is not obvious, it is more likely that the differential expression of these miRNAs alters KIF5B expression and impairs dopaminergic synapses, leading to PLE. Additionally, experimental evidence for hsa-miR-142-3p, hsa-miR-199a-3p, and hsa-miR-486-5p in the regulation of ITGB8 is available^63–65^. ITGB8 is a member of the integrin beta chain family, which plays an important role in neurovascular development, synaptogenesis, synaptic stabilization, and maintenance by regulating ECM interaction^66–68^. These synaptic changes caused by ECM abnormalities are often observed in schizophrenia^44^. Thus, the differential expression of hsa-miR-142-3p, hsa-miR-199a-3p, and hsa-miR-486-5p might be responsible for PLE via ECM abnormalities. Investigating the function of these pathways may enable us to determine the biological mechanisms underlying the transition from persistent PLEs to these disorders.

This study had some limitations. First, the sample size was relatively small. However, we additionally used alternative statistical methods (the Benjamini–Hochberg method and cross-validation) to obtain reliable and accurate results. Our results should be validated using independent cohort samples. Second, it was difficult to evaluate the physiological and functional associations between urinary exosomal miRNAs and PLEs. This is because urine biopsy remains largely unexplored in psychiatric research, and its functional significance remains unknown. Thus, further functional experimental studies are required. Third, our evaluation of the predictive model constructed for the six miRNAs was limited to a predictive performance for only 1 year. The performance over longer periods can be evaluated using the follow-up assessments of PLEs in the Tokyo Teen cohort.

In conclusion, this study identified six miRNAs in urinary exosomes that were significantly altered in adolescents with persistent PLEs. These miRNAs (hsa-miR-486-5p, hsa-miR-199a-3p, hsa-miR-144-5p, hsa-miR-451a, hsa-miR-143-3p, and hsa-miR-142-3p) could predict the trajectory of PLEs after a year with high accuracy using the logistic regression model.

Thus, despite the small sample size, this study suggests that urinary exosomal miRNAs in adolescents are a novel biomarker for assessing the risk of psychiatric disorders.

## Supplementary information

Supplemental Material

## Data Availability

The data that support the findings of this study are available upon reasonable request from the corresponding author, M.A.
