# Supplementary material for: Urinary exosomal microRNAs as predictive biomarkers for persistent psychotic-like experiences
Source: Schizophrenia (Heidelb). 2023 Mar 11;9(1):14. doi: 10.1038/s41537-023-00340-5 (PMC10008540; doi:10.1038/s41537-023-00340-5)
Supplement: Supplementary file 1 — Supplemental Material [file 41537_2023_340_MOESM1_ESM.docx]

**Supplemental Material**

**Table S1: Differentially expressed microRNAs between the persistent and remitted groups**

|  | logFC | logCPM | *P*-value | FDR |
| --- | --- | --- | --- | --- |
| hsa-miR-486-5p | −4.284 | 9.898 | 1.882E-06 | 0.000805 |
| hsa-miR-199a-3p | −2.966 | 7.899 | 4.675E-06 | 0.000920 |
| hsa-miR-144-5p | −5.567 | 3.337 | 6.449E-06 | 0.000920 |
| hsa-miR-451a | −5.111 | 8.313 | 1.233E-05 | 0.00132 |
| hsa-miR-143-3p | −2.917 | 6.399 | 0.000120 | 0.0103 |
| hsa-miR-142-3p | −2.785 | 7.156 | 0.000335 | 0.0239 |

Small RNA-seq analysis revealed six differentially expressed urinary exosomal miRNAs. FDR is adjusted *P*-values using the Benjamini-Hochberg false discovery rate method. FC is the fold-change (persistent vs. remitted). CPM is the average count per million among the participants. FDR < 5% and changes greater than two-fold were considered statistically significant.

**Table S2: Enriched pathways associated with MDD**

| KEGG pathway | Adjusted *P*-value | Reference |
| --- | --- | --- |
| Thyroid hormone signaling pathway (hsa04919) | 1.38E-05 | (Zhou Y, 2021) |
| Sphingolipid signaling pathway (hsa04071) | 0.0165 | (Zubenko GS, 2014) |
| Estrogen signaling pathway (hsa04915) | 0.0323 | (Zhao Y, 2020) |
| ErbB signaling pathway (hsa04012) | 0.0323 | (Carboni L, 2018) |
| mTOR signaling pathway (hsa04150) | 0.0323 | (Jerigan CS, 2011) |
| Dopaminergic synapse (hsa04728) | 0.0323 | (Liu Y, 2019) |
| TGF-beta signaling pathway (hsa04350) | 0.0471 | (Musil R, 2011) |

Abnormalities in seven pathways among the enriched pathways of the six miRNAs reported in MDD. KEGG: Kyoto Encyclopedia of Genes and Genomes; MDD: major depressive disorder; miRNA: microRNA.

**Figure S1: Comparative analysis of the urinary exosomal miRNA in adolescents between those with PLEs and without PLEs.**


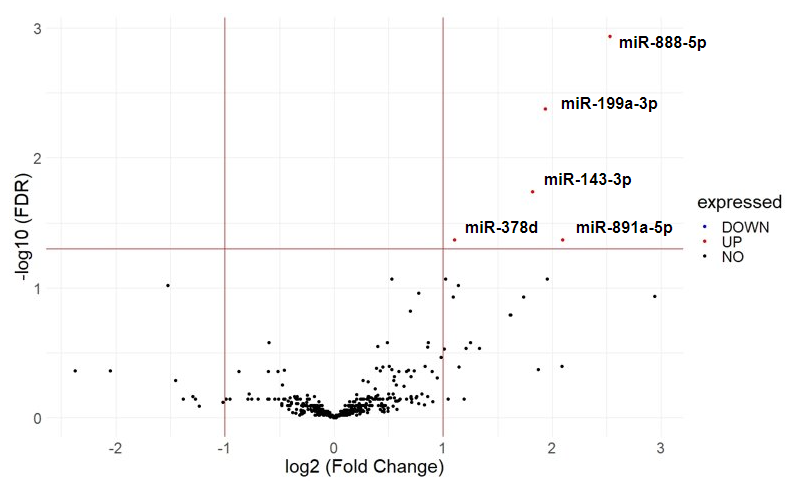
Comparison of miRNA expression between individuals without and with PLEs at baseline. The fold change (case vs. control) of each miRNA is plotted against its p-value; red circles represent miRNAs with FDR < 0.05 and log_2_FC > 1.0.
